# “Truly Listen to Us”: Recommendations for Health Professionals to Bolster Wellbeing of Nonbinary Individuals

**DOI:** 10.3390/ijerph19159032

**Published:** 2022-07-25

**Authors:** M. Killian Kinney, Darren Cosgrove

**Affiliations:** 1 Claire Argow Social Work Program, Pacific University, Forest Grove, OR 97116, USA; 2 Department of Family Science & Social Work, Miami University, Oxford, OH 45056, USA; cosgrod@miamioh.edu

**Keywords:** nonbinary health, trans-affirming healthcare, healthcare providers

## Abstract

Trans-affirming providers play significant roles in the health and wellbeing of nonbinary individuals. Yet, healthcare mistreatment is well-documented among gender-diverse patients, leading to clients withholding information and avoiding care for fear of experiencing bias. Concurrently, healthcare providers report feeling ill-equipped to serve nonbinary patients, often perpetuating cisnormative binary attitudes. The literature has established the challenges to accessing healthcare and the need for gender-affirming care. However, little is known about nonbinary people’s perspectives on how best to deliver gender-affirming care that is inclusive of nonbinary patients. This participatory action PhotoVoice study identified community member recommendations for healthcare providers to bolster the wellbeing of nonbinary individuals through improved access to gender-affirming healthcare. Data were collected through group discussions, photography, and photo-elicitation interviews. Drawing upon research results, the authors identify recommendations for improving interpersonal care, increasing access to gender-affirming care, and advocating for related environmental and policy changes.

## 1. Introduction

The interaction between ones’ gender identity and expression, and ones’ environment plays a critical role in nonbinary people’s access to mental and physical healthcare, and related biopsychosocial outcomes. Cisnormativity, transphobia, and binarism (the assumption that two genders – man and woman – are the only genders and, thus, social systems are built for these two genders, erasing and excluding gender expansiveness) social gender paradigms all contribute to disparate adverse health outcomes for trans and nonbinary people [1,2,3]. As trans and nonbinary people navigate transphobic sociopolitical factors contributing to increased rates of housing instability, unemployment, violence, and avoidance of preventative care, there is a marked need for access to affirming and culturally-responsive services that can both address the complicated impacts of discrimination as well as promote (self and ally) advocacy that advance community wellness. Moreover, while a great deal of trans-focused research and healthcare delivery (including mental health services) has previously focused on the diagnosis and treatment of gender dysphoria, we are presently in the midst of a professional paradigm shift. More and more, scholars and practitioners are moving away from the deficit-based assessment of individuals and towards acknowledging the significant role that society’s lack of acceptance plays in contributing to adverse biopsychosocial outcomes. 

The present study aimed to engage nonbinary participant researchers to identify factors in their lives that contributed to or diminished their wellbeing and how this information could be used to improve their lives and the lives of nonbinary communities, of which the healthcare-focused results have been reported in this manuscript. In doing so, this work centered on lived experiences and nonbinary leadership while also building scholarly knowledge that supplements the abundance of information about adversity and health disparities. Though the prevailing theories of wellbeing, namely subjective and psychological wellbeing, informed the study, they do not provide a comprehensive conceptualization that accounts for marginalization [4]. In the absence of a fitting wellbeing theory, the concept of wellbeing among nonbinary individuals was defined as a state of thriving in which a person’s basic needs are met, and they are able to comfortably and safely be themselves and pursue their goals in their current environment, in which thriving was characterized by a positive self-identity, satisfaction and fulfillment in life, and conditions that help people achieve their goals [5].

There has been growing attention to trans identities and experiences within medical and mental health literature [6,7]. While this trend is a promising move towards greater awareness of gender diversity and the needs of trans people, there has been relatively little attention paid to the specific experiences and needs of those who are nonbinary [6,8,9]. Nonbinary is a broad umbrella term for people whose gender falls outside of or does not fit exclusively within a dichotomous man-woman paradigm [10,11]. Although not all nonbinary people identify as transgender, a 2015 U.S. national study found that, at 35%, the category nonbinary made up the largest subpopulation of the survey’s nearly 28,000 respondents [12]. While there is an under-representation of nonbinary-focused literature, what has been noted is that nonbinary people may face greater rates of discrimination and psychological distress than their binary trans peers [8,13,14]. Moreover, such discrimination has been associated with a range of psychological, social, and physical health risks [12,13,14].

This study partnered with nonbinary research participants to explore factors that contribute to their wellbeing. Adopting the participatory action research (PAR) methodology PhotoVoice, this study engaged nonbinary adults ages 18 to 50 in an arts-based inquiry focusing on factors that supported or served as a barrier to wellbeing. A broad study aim was presented to allow for collaborative exploration with study participants. Throughout this engaged research process, participants reflected on their individual and shared experiences, analyzed qualitative data, and developed results that elucidated factors that deteriorate and improve their wellbeing. We present these results with the hope that they contribute to a holistic understanding of nonbinary people’s diverse gender-affirming needs so that scholars and providers can not only work toward eliminating harmful discriminatory practices but also, promote those that facilitate individual and community wellbeing. 

### 1.1. Trans and Nonbinary Healthcare

Access to trans-affirming healthcare is a social determinant of health and should be prioritized as such by healthcare providers, healthcare systems, and society [15]. Unfortunately, national data suggests many gender-diverse individuals experience barriers *to* and discrimination *within* healthcare settings [12,16]. Such discrimination has resulted in gender-diverse people being considered a medically underserved population [17,18]. Moreover, a review of social science literature finds that many trans and nonbinary people have experienced gender victimization and anti-trans traumas, and experiences of hostility in healthcare settings [19]. 

Although clients across diverse trans identities routinely experience inadequate healthcare, Frohard-Dourlent et al. [20] argue that, within healthcare systems, there exists a privileging of binary transgender identities (i.e., trans men and trans women) over those that are nonbinary. This is suggested due to pre-existing medical and social constructs about what it means to be trans and about gender itself. More specifically, it is often erroneously assumed being trans is necessitated upon a linear path of gender transition *from one side of a dichotomous binary to the other*. Consequently, as long as healthcare policies are written within binary-centric paradigms, nonbinary individuals will continue to experience barriers to gender-affirming care [15].

### 1.2. Cisnormativity and Erasure in Healthcare

Trans and nonbinary people require the same diverse healthcare services that cisgender clients do; however, such care must be provided in ways that are responsive to and understanding of their gender and the unique experiences they may face in a cisnormative and binaristic world. Additionally, many trans and nonbinary people may seek gender-affirming medical care to help their bodies better align with and express their gender [12,21,22]. More germane to the focus of this paper is that many trans and nonbinary people may experience a greater need for accessible and comprehensive healthcare services, given the staggering health disparities faced as the result of ongoing discrimination. Nonetheless, cisnormativity and trans-erasure within health systems result in care frequently becoming illusively unattainable, sub-par, or actively harmful.

Author and scholar Viviane Namaste [23] describes the concept of trans-erasure as a social, cultural, and institutional nullification of trans identities to the point of impossibility. Such erasure is pervasive throughout much of the social fabric of the U.S., and thus, unsurprising that broad social erasure has found its way into the realm of healthcare. In healthcare systems and settings, trans-erasure can manifest both institutional and informal erasure. For nonbinary service recipients, informal erasure of identity often takes the form of providers being unprepared to understand their identities or health needs, whereas institutional erasure involves a lack of inclusive paperwork and policies [24]. For nonbinary people holding multiple marginalized identities and navigating the intersecting systems of ablism, racism, heterosexism, and cissexism, there may be an experience of identity bifurcation as service users navigate decisions regarding which identities, they feel safe sharing with providers and which they do not [8]. 

Cisnormativity compromises trans and nonbinary healthcare through both direct and indirect means. Health is compromised indirectly through trans people’s disproportionate lack of health insurance (often the result of unstable employment [12,15] and policy barriers that prohibit the acquisition of care [21,22]. For many trans people who seek gender-affirming medical care, gatekeeping in the form of required mental health assessments and referral letters is seen by many as an additional and pathologizing service system hurdle that must be cleared in order for care to be delivered [25]. When such indirect barriers are navigated and one finds themselves in a clinic or other healthcare settings, more direct challenges may arise, including provider-based discrimination such as physical mistreatment and denial of needed care [12,26]. Moreover, trans and nonbinary people frequently report that when healthcare services are accessed, non-inclusive paperwork, intake assessments, and a lack of knowledge about trans identities, trans bodies, and trans health result in stigmatization and pathologizing experiences that further marginalize clients who may already be disenfranchised from the healthcare system [9].

### 1.3. Gender-Affirming Healthcare

Trans-affirming healthcare providers play a significant role in mental health for trans and nonbinary individuals [27]. Although many clients may experience difficulty finding a trans-affirming provider or a provider who offers services related to medical transitioning [24,28]), those with transgender-affirming primary care physicians are reported to be eight times more likely to have pursued a medical intervention than those without [29]. While access to care is crucial, many healthcare practitioners report feeling ill-equipped to serve the trans and nonbinary community [30] and reflect cisnormative attitudes due to a lack of trans-informative education [15].

Approximately 24% of the 9,700 nonbinary participants engaged in a large 2015 national survey of trans people, reported negative experiences with healthcare providers [12]. A previous report, drawing data collected from a prior national trans-focused survey, found nonbinary respondents noted fewer reported medical refusals due to bias (14.0%) compared to the overall sample (19.0%); they reported higher postponement of needed medical care due to fear of experiencing bias (36.0% compared to 28.0%) [14].

Nonbinary individuals who have experienced discrimination from medical professionals may withhold information or postpone/avoid care [1,31,32] and at rates more frequent than their cisgender LBG peers [33]. Compared to their binary trans peers, nonbinary people have reported less physical and mental healthcare utilization (despite increased need) [34]. Fear of negative experiences in healthcare settings has led some trans and nonbinary individuals to self-treat (e.g., hormones) [24]. In other instances, those seeking care may attempt to “pass” as cisgender with the hope of avoiding discrimination [15].

With the emergence of the World Professional Association of Transgender Health (WPATH) Standards of Care (SOC), guidelines have been established with the intent to guide practitioners in providing gender-affirmative care [35]. The most recent version of these standards (SOC 7) included language that was changed from binary-centric to nonbinary-inclusive Moreover, these standards include a statement recommending practitioners not impose the gender binary on youth [35]. These modifications evidence an increasing professional awareness of nonbinary identities and an established need for more inclusive practices [20]. Similarly, the Gender Affirmative Model (GAM) was introduced in 2013 and recognized gender diversity as not disordered and present across cultures and considered the environmental context and its impact on gender [36,37]. Recently, Rider et al. [38] published the gender affirmative lifespan approach (GALA), a psychotherapeutic framework for competent tailored clinical care specifically for nonbinary individuals. The GALA model’s five core components to affirm nonbinary clients are: (1) building resiliency; (2) developing gender literacy; (3) moving beyond the binary; (4) promoting positive sexuality; and (5) facilitating empowering connections to medical interventions (if desired) [38].

### 1.4. Nonbinary Access to Healthcare

In addition to navigating many of the aforementioned challenges that binary trans people face, many nonbinary people report unique barriers in their attempts to access care [1]. Such barriers are often connected to binarism (or paradigms held by providers that are binary-centric) [1,8]. A primary example of binarism is health insurance policies for top surgery (top surgery is a type of mastectomy with flattening or masculinization of the chest for AFAB individuals or a mammoplasty to feminize the chest for AMAB individuals [39]) which increasingly more nonbinary individuals are seeking [39]. Gender-affirming surgery policies are frequently written exclusively for transitioning from male to female or female to male. While not represented in the literature yet, nonbinary people have experienced insurance claim denials with policies that are designed with binaristic language (e.g., the need to transition to *the other or opposite* gender), which can be used as grounds for denial when their claims and professional support letters do not reflect binary language. For those who identify outside of the gender binary, this well-intended policy is far from inclusive. Recent strides have been made to expand healthcare policies to include gender diversity. The CG-SURG-27 policy listing requirements for gender-affirming surgeries used by BlueCross BlueShield and other large insurance firms was updated in 2018 to change the language from “the individual is a female transitioning gender to become a male” [40] to read “the individual is a female desiring gender transition” [41]. This simple change in language has powerful implications, and it serves as an example for the further critical assessment of current and new policies to prioritize the elimination of barriers to care for nonbinary individuals.

### 1.5. Significance of the Study

Nonbinary individuals have been identified as a marginalized population in the literature and, therefore, germane to social justice and equity-focused healthcare research and practice. Social justice for this population includes health equity, legal representation, and social inclusion, among others. Regardless of the specific service field (medicine, mental health, epidemiology, law, etc.), practitioners will have clients or work alongside individuals who are trans and nonbinary in their careers [42]. Without increased cultural awareness and comfort working with gender diversity, practitioners may inadvertently cause more harm than good. The literature supports the need for gender-affirming care to reduce barriers and improve health outcomes for trans and nonbinary individuals. Despite unique experiences among nonbinary individuals, gender-affirming care recommendations are not provided that explicitly consider nonbinary experiences. 

The study was developed to build upon the current health and human service literature focusing on the needs of trans and nonbinary people. Moreover, this work aims to bring unique attention to the specific needs and experiences of nonbinary clients. Through the use of arts-based participatory action research, we center on the lived experiences of a group of nonbinary young adults and draw upon the reflective meanings they assign to their experiences as we offer healthcare practice recommendations. We note opportunties for providers working with nonbinary community members to intervene at multiple levels of social ecology to bolster wellbeing.

## 2. Materials and Methods

This study sought to contribute to a better understanding of wellbeing among nonbinary individuals in an effort to mitigate the challenges they face as well as support thriving despite the binary world in which they exist. Seeking to build knowledge while also fostering social connectedness and change, the first author engaged the PAR methodology PhotoVoice to explore experiences related to wellbeing among nonbinary individuals, including promotive and corrosive factors of wellbeing. Approval was obtained from the Institutional Review Board at Indiana University.

### 2.1. Participatory Action Research 

PAR was developed as an approach to knowledge-building that aims to actively advance social change and justice in ways not commonly found in other forms of research [43,44]. Notably, PAR asserts that those most impacted by a topic should be central in understanding it [43]. Moreover, it is in the act of “doing” or taking action that experiences can be further understood. Consequently, PAR typically involves a series of iterative cycles of taking action, observing, and reflecting [45].

Two distinct approaches to PAR have emerged—the “northern” and the “southern” tradition [46,47]. Emerging from Kurt Lewin’s [48] theory of action research, the “northern” tradition took root in the northern hemisphere and embraced the idea of “knowledge for action” while paying attention to the often disconnected dynamics between researcher and subject, and typically focused on organizational change [44,47]. The “southern” tradition, on the other hand, emerged as the “radical” tradition and is grounded in the work of Paulo Freire [49,50] and the political movements of the southern hemisphere of the 1960s and 1970s [46,47]. Radical PAR seeks to critically examine social and political realities and problematizes positivist notions of knowledge generation and truth. Moreover, PAR is distinguished from other research methods as it shifts dynamics between researcher and participant. Moving the research relationship away from data *collector* and *giver* to a reciprocal educational relationship that strives for consciousness-raising about social realities [51,52].

PAR has been specifically identified as a method for studying social constructs and power, including gender and its attendant power dynamics [53]. PAR does not ignore the social and historical context of the individuals and their experiences but rather situates the variables within power and privilege [53]. As such, the first author felt it was an ideal methodology for the exploration of cisnormativity and the lived experiences of nonbinary adults. Considering the lack of visual social representation and erasure reported by nonbinary people [24,54,55] and the likely influence of gender visibility on wellbeing, PAR combined with the use of visual methods is a fitting approach to explore wellbeing with the nonbinary community. 

### 2.2. PhotoVoice

One methodological approach to PAR is the arts-based method of PhotoVoice. First developed by Wang and Burris (1997), PhotoVoice is a PAR method that is both accessible and flexible and whose processes can be of benefit to participants [56]. According to Wang and Burris [57] PhotoVoice is grounded in empowerment education, documentary photography, and feminist theory see [58,59]. As an arts-based method, PhotoVoice is well-fit for exploring abstract social constructs, such as gender and wellbeing, while producing abundant and meaningful data [60]. The method positions community members as co-researchers who document their lived experiences through photographs. Photographs are then shared with the other participant researchers and collectively analyzed. Typically, the group’s photographs and results are then shared with community stakeholders as a tool to advocate for community change related to the topic of inquiry [57,61,62].

PhotoVoice has been used to examine and expose social justice topics and promote action to improve the identified conditions [58,63,64]. Previously, PhotoVoice has been used to explore identity meaning-making, health and humans service barriers faced by nonbinary young people [8,9], the experiences of LGBTQ young people formerly in foster care [65], and the experiences of LGBTQ college students [66]. Others have suggested that this methodology can be useful in gender and sexuality identity exploration [67], and Ingrey [68] goes as far as to describe photovoice as a genderqueer methodology for its ability to critically deconstruct gender and gendered spaces and pursue gender justice. 

#### Virtual Adaptation

Data collection and group-based analysis for this study occurred between June and August 2020. Given that the world was amid the global COVID-19 pandemic during this time, it was necessary to adapt the PhotoVoice methodology as in-person group meetings were not safely feasible. Consequently, the first author designed the study to occur virtually through online platforms to conduct group-based work. Canvas, a secure cloud-based Learning Management System (LMS) developed by Instructure Inc. (Salt Lake City, UT, USA), was the platform used for this virtual study [69]. To protect the privacy and security of information, all communication on Canvas uses an HTTPS address with all inbound and outbound traffic encrypted and mechanisms for identity authentication, automatic updates with security patches, and physically secure servers [69].

### 2.3. Recruitment and Sampling

Digital flyers promoting the study were distributed among community leaders, community organizations, and those working with transgender and nonbinary individuals to promote convenience and snowball sampling. Individuals were eligible to participate in the study if they identified as a gender that was not exclusively a man or woman, were aged 18 years or older, lived in the Midwestern United States (North Dakota, South Dakota, Nebraska, Minnesota, Iowa, Missouri, Wisconsin, Illinois, Kansas, Michigan, Indiana, and Ohio), and had the technology to engage in a virtual study. Compensation for participation in this study mirrored previous PhotoVoice studies [70,71], providing a USD 10 gift certificate per data collection activity. Informed consent was obtained before starting the study. Additionally, before photos were collected, participant researchers (PR) signed a photo release form that identified how they permitted their photos to be used in various study-related activities and dissemination. A sample of 24 participants was recruited and a final sample of 17 participants completed the study, which is a similar sample size to other PhotoVoice [61,70,71]. As this was run as two groups (groups of 12 that concluded with 8 and 9 members), this fits with Wang’s [62] recommended 7 to 10 people to be the ideal number of participants for a PhotoVoice study for practicality and in-depth discussions. 

### 2.4. Data Collection and Analysis

Data were collected by the first author using asynchronous Canvas group discussions, through participant researcher (PR) photographs, and photo-elicitation interviews conducted over Zoom. Additionally, reflexive memoing was recorded by the first author throughout the study. PRs engaged in several online discussions as they shared and analyzed their photographs. This article draws upon data shared in one such discussion that asked PRs to reflect upon the group’s photos and develop health and human service practices and policy recommendations. Specifically, through this facilitated discussion, PRs responded to the following prompts:

1. What emerging themes do you hear as we have discussed these priorities for change to promote wellbeing in our lives and the lives of our communities?
2. How would you recommend the information about wellbeing (e.g., promotive and corrosive factors) applied to practice with nonbinary communities to bolster wellbeing?
3. How would you recommend the information about wellbeing (e.g., promotive and corrosive factors) applied to policy with nonbinary communities to bolster wellbeing?

In addition to engaging in group dialogue, PRs analyzed their photographs in-on-one photo-elicitation interviews with the first author. Interviews explored promotive and corrosive factors to their wellbeing, including gender-affirming and disaffirming experiences with providers, healthcare systems, and insurance companies.

Visual data and transcriptions were analyzed by the first author using thematic analysis, which followed [72] initial coding, focused coding, and axial coding. A codebook was created collaboratively with a cisgender heterosexual colleague, who provided an outsider perspective to increase rigor and trustworthiness. Emerging themes were discussed with participants. Additional mechanisms for trustworthiness included reflexive analytic memoing and consultation throughout data collection and analysis.

## 3. Results

The results will be presented in two categories. First, PR narratives focusing on personal healthcare experiences will be presented to provide a more nuanced understanding of nonbinary perspectives. Then, recommendations from PRs for improving gender-affirming healthcare will be discussed within the context of three levels—individual, organizations, and systemic change.

### 3.1. Sample

The study sample was diversely represented in age, body type, disability status, and neurodiversity but not by race (Table 1). All but one PR identified as white for race (93.8%). Nuances emerged when discussing ethnicity and culture. Culture was shared in relation to their people (Oglala nation, Taíno Puerto Rico, first-generation immigrant European American, disabled), identity (queer, areligious, Jewish—not practicing), geography (Midwestern, Midwest transplant from the deep south originally, rural), and history (adopted, “raised Hungarian—pure American—watched (their Hungarian culture) disappear”, “orphaned by our own culture…seek out our own soul”). The sample was skewed toward those with college experience and degrees. Most PRs had earned a bachelor’s degree or higher (58.8%), and 23.6% were current college students. PRs lived across the Midwest, including Indiana (*n* = 5), Michigan (*n* = 4), Ohio (*n* = 4), Missouri (*n* = 2), Illinois (*n* = 1), and South Dakota (*n* = 1).

### 3.2. Healthcare Experiences

PRs reported many challenging experiences when accessing healthcare services. The following narrative from HG (PRs provided the name or label they wished to share in publication, which included initials, chosen names, and simply a number for one participant) articulates many of the similar experiences that were shared by the group. Of note, the common experience PRs had was that accessing healthcare can either be a painful and dysphoria-triggering experience or an incredibly affirming one depending on the provider.

*So this one can be kind-of triggery…I’ve had really bad experiences particularly with gynecological health…my hormones are not standard…I tend to run low on estrogen, high on testosterone…[providers] try hormone therapies and they try this and they try that and “oh, we don’t understand why that’s painful and like”—or “that’s just how it is for some women, you should, you know”.*

**Figure 1:**
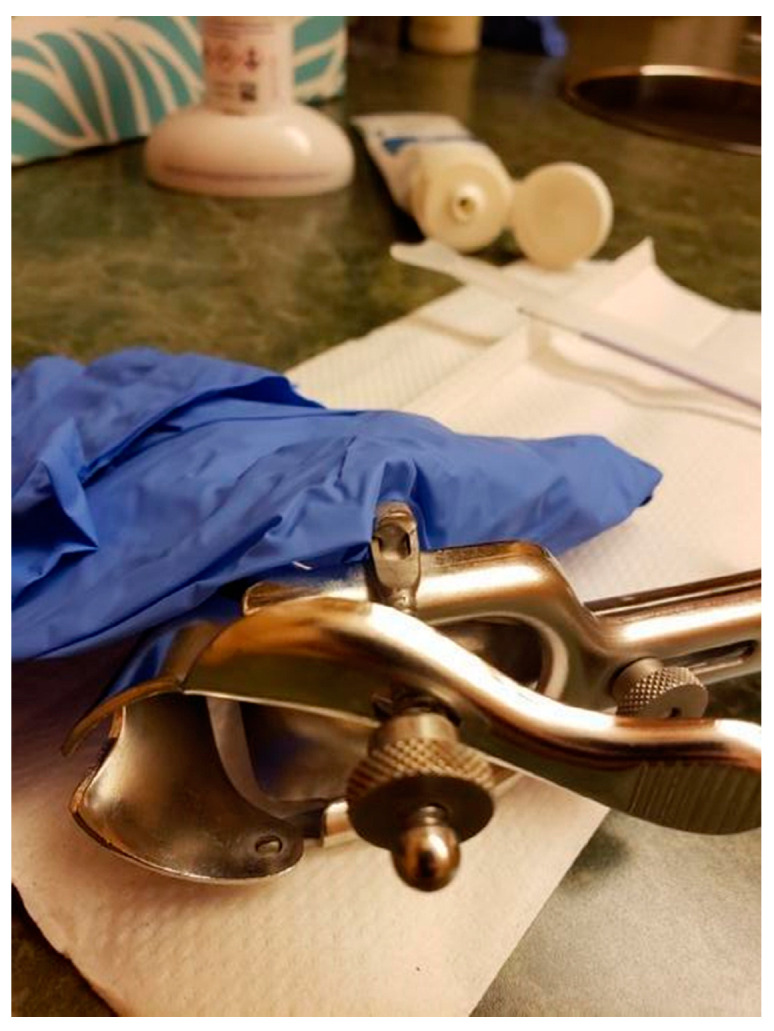
“Health Care—It’s Complicated” (HG).

*Contrasted with my current situation where I have a very affirming gynecological practice and a doctor I trust and they make every effort every time I go in to make me as comfortable and [be] as affirming as possible. They’re willing to talk to me about the fact that I have female partners, they’re willing to talk to me about the fact that I’m poly* [Polyamorous, which is a relationship structure characterized by multiple consenting romantic and/or sexual relationships]. *They’re willing to talk to me about consent-based STD [sexually transmitted diseases] and STI [sexually transmitted infections] testing and they’ve never shamed me for it…When I started seeing her there was some education. She asked questions and she listened, and so when she asked for resources, she’s like “oh, do you have anybody you would recommend reading?” And I gave her a couple of books. Six months later when I went in for another checkup, she had read them and she’d looked at them and remembered. Now when I go in, like it’s in my file and people in the practice know and continue to affirm [me]. At one point they changed their intake forms and they’re like “Will you look at our new intake forms and see if you would add anything and they added a line for, you know, your legal name? And then they added “what would you like us to call you in the office” as a line. They added multiple genders and were welcoming people to define for themselves. It makes me so much more appreciative of having a medical care provider who is compassionate and who is dedicated to at least educating themselves…above and beyond just being affirming, they’re being proactive and that’s been a game changer.* (HG) (see Figure 1)

In this narrative, HG identifies ways they felt seen, respected, and affirmed by their provider. They did not feel judgment or curiosity but rather an openness to help meet their needs, including self-motivated learning. These characteristics are examples of affirming indicators that can foster trust and comfort with trans and nonbinary clients—and are, arguably, a universally affirming approach.

In addition to stigma based on gender, PRs noted their intersectional identities and other forms of mistreatment from medical providers. Many PRs discussed the issue of fatphobia in social and medical contexts as a significantly detrimental challenge. Tristan identified the gap of fat activism in queer and trans space and how this creates a barrier to trans-affirming surgeries:

*I really need queer and trans people to just listen to me, say that it sucks being limited because of my fatness without telling me that being skinny is hard, too. I’ve been really moving away from body positivity narratives in spaces recently and more towards fat liberation politics because I think that body positivity feels nice and inclusive, [but] it never actually does anything for me—I just feel more shitty about myself because I can’t make myself love my body. But I think that a fat liberation channels anger better. Like why is it that fatness precludes people from practicing medical transition—that’s actually completely arbitrary. The way that fat phobia and transphobia combine in medical settings has caused real serious problems for me that aren’t always even just about transition care, but that are just about trying to get regular healthcare from a doctor as a fat trans person is not possible…and so yeah…I need people to get on board with fat liberation.*

Tristan’s narrative provides a passionate description of the need for intersectional activism in healthcare to mitigate the erasure and harmful exclusion of trans and nonbinary individuals. As Tristan stated, people need to have access to trans-related care as both nonbinary and fat individuals, without the latter precluding the former. Cory spoke of anxiety around facing fatphobia when pursuing top surgery:

*The surgeon’s office technically has a BMI [body mass index] restriction and so I’m chubby…I’m nervous because the clinic didn’t tell me anything and I’m assuming that they would think I’m fine or would have told me if I’m not fine so I’m in this weird—“am I gonna be fat-shamed by the medical field today?”*

Thus, searching for a reputable surgeon to reduce the chance of being fat-shamed and refused top surgery is necessary. Sizeism was one of several needs for intersectionality in healthcare, which will be discussed in greater detail in a later section.

### 3.3. Accessing Healthcare

When asked about desired outcomes to bolster their wellbeing, accessible and affirming healthcare was a frequent response from PRs. Timothy identified high priorities as “access to comprehensive, affordable, and inclusive healthcare options” and, ideally, “having access to a community-focused, LGBTQ+ [lesbian, gay, bisexual, transgender, queer] run health clinic”. Timothy’s concerns seemed to resonate with other PRs and several noted that, when available, they transferred care to their local trans-focused health clinics. Cory shared the following,

*I switched my ob/gyn to the trans clinic because—well, the one that I had was very…you know, “you’re a girl and you want to have kids” and I’m like I’m just here to check off the box and be done. I’m feeling really dysphoric and uncomfortable with you rather than recognized for my gender and that type of thing…It is frustrating, but it’s also definitely invalidating because you have to sit through a very uncomfortable situation anyway because nobody really wants to be at the doctor and misgendered. It’s like I’m already not feeling well, you have to kick me while I’m down, too?*

Cory continues to describe the importance of gender clinics within geographic and cultural contexts:

*In the Midwest where transgender clinics are not as common or Planned Parenthood [a common provider of gender-affirming OBGYN and hormone care] is being defunded, that type of thing and so having spaces for us to get healthcare without having to revert back to our assigned gender or having to explain to your doctor.*

Despite agreeing that all healthcare should be changed to become more gender-affirming, there was consensus that PRs sought out specifically gender-affirming providers when available for their own personal safety, comfort, and health.

Outside of the trans-specific healthcare settings, PRs described their experiences as wrought with numerous challenges, such as microaggressions of deadnaming (deadname is the label adopted by some trans and nonbinary people for the name given to them – in contrast to the name they chose for themselves and being deadnamed is the act of being called by this former name when they no longer use it with responses to this act ranging from discomfort to triggering trauma), misgendering, and inappropriate curiosity-based questions. Noting the burdensome nature of these challenges, Cory commented, “[It is] a lot of hurdles that it adds just to be affirmed”. Providers could both be a source of trauma as well as play a role in healing past traumas, depending on the provider and whether they were gender-affirming (and arguably equipped to identify and address trauma). Gender-affirming therapists and mental health providers were highly valued among PRs, such as HG’s narrative of compassionate annual care with intentionality to reduce dysphoria that exemplifies the potential for healing within triggering circumstances. In contrast, non-affirming providers were identified as sources of trauma and delays in identity development.

PRs reported avoiding necessary care out of fear regarding the gender-specific microaggressions they anticipated experiencing. Cory shared that they found themselves “skipping pap smear and other care due to not having affirming providers” and Jynx commented that a coping response they used was “never showing up to doctor’s appt [even for serious medical concerns] because of a lack of trust”. Jynx continued bluntly, “it shouldn’t take somebody putting themselves potentially at risk [to receive affirming care]”. Avoidance of care due to minority stressors poses a significant public health concern for nonbinary populations that could be mitigated by gender-affirming providers.

### 3.4. Recommendations for Healthcare Providers

As PRs engaged in the virtual PhotoVoice process, they gave consideration to their own individual works (photographs and narratives) and the larger body of work produced by the collective group. In reflecting on the salient themes present in the data, PRs considered ways in which the acquisition and delivery of healthcare could be increasingly affirming for nonbinary service recipients. Collectively, PRs identified strategies for healthcare providers and systems, which are organized by *Recommendations for Improving Interpersonal Care, Recommendations for Increasing Access to Gender-Affirming Care,* and *Recommendations for Advocating for Environmental and Policy Changes.* Because participant researchers had experienced exclusion and invisibility, they eagerly shared their experiences, both of support and challenges, which informed their recommendations. At the heart of these recommendations is a call for advocacy—self-advocacy, organizational advocacy, policy advocacy, and social advocacy.

### 3.5. Recommendations for Improving Interpersonal Care

**Do the Self-Work.** PRs wanted cisgender providers to recognize their gender-based privilege and reflect on their role in the perpetuation or disruption of transphobia and marginalization so that they can assess ways to improve. In particular, PRs emphasized self-reflection on gender and gendered practices—“I’m not sure how [their photo] could educate others [other] than to have people self-reflect on the way that they take care of themselves and if they run into any weird gender feels” (Kristy). Assessing feelings was said to be indicators of when to check one’s biases and assumptions about gender diversity, particularly what a nonbinary person looks like, to avoid invalidating someone who does not conform to expectations. “I think that [providers] need to understand that they have to get rid of their pre-conceived notions…the nonbinary experience is very unique to every person” (Nat). Nat reported having their gender invalidated by others, which they attributed to their socially nonconforming gender expression (SNCGE). “Just because I’m very feminine in my trans masculine nonbinary existence doesn’t mean that that’s the case for everyone. Just because I’m nonbinary transmasc doesn’t make me any less theoretically masculine than a binary trans person” (Nat). According to Jynx, acceptance requires all of us “...to look at our bias (implicit or not) and counter the harmful barriers we’re putting on ourselves and others”. Further elucidating this point, Dylan provided the analogy of a blank slate was given for how they would like people to engage with them:

*I want to just navigate the world as me and be seen for me and no automatic assumptions based on my voice or my breasts or my body shape or anything like that—it really is just a blank slate in some way of just being able to then show the world who I am.*

This blank slate approach includes asking open-ended questions within healthcare contexts, such as “How would you like me to refer to you in our meetings?” or “Do you have any sexual partners”. Open-ended questions can reduce assumptions and allow individuals to divulge the information they feel comfortable sharing at that time. 

**Continue to Educate Oneself.** Providers who educated themselves on nonbinary gender identities, expressions, and experiences were important for PRs’ wellbeing, particularly among mental health providers. PRs agreed upon the importance of having a knowledgeable and affirming therapist, as well as the challenge of finding one. For those who had an affirming therapist with whom they could discuss gender, the benefit was significant. HG described this experience as:

*The difference in the internal reality and how then I interact differently with the world because I can interact from a place of sovereignty and power and self-assurance—I like who I am and I like how I interact with people better…much more comfortable with boundaries and like “these are for my wellbeing, please don’t cross them”.*

In order to obtain the benefits of therapy, some PRs had to educate their therapists. Catkin recalled an awkward start with a new therapist but “was really impressed this last time because she looked up nonbinary and was reading about it, and I thought this is a good sign because my first meeting went…all awkward and weird, but the second one went really well”. PRs fully accepted that people make mistakes, but these blunders varied by how others responded and whether an effort was perceived. Noel affirmed this by explaining, “I just want to see that you’re trying to respect who I am…and you’re willing to learn from the mistakes and that you’re willing to educate yourself”.

An important distinction of the self-work process was educating oneself through various sources that did not require the emotional labor of nonbinary clients. The emotional labor of educating others has been established as a point of significant exhaustion for nonbinary individuals, especially when an abundance of information is available online. Another perspective was for people to educate themselves through exposure to nonbinary people. Or, as Noel stated, “Don’t just Google!” and further expanded:

*I think that a lot of times when someone says “ok, I need to learn about how to be better to queer people”—they just Google it and are just like “what is queer?” Ok, “what is they/them?” So we have this very sanitized way of learning…but I think it’s so much important to listen to individual people—their voices and their experiences and to find that shared humanity because I think when you find that shared humanity—that puts you in a place where you really do feel in your heart that you want to help ensure that our wellbeing is good.*

Some PRs reported being willing to assist providers who want to learn—“If you want to understand it, cool. We’ll find you all the resources” (Jynx). While some PRs expressed a willingness to educate well-intentioned others, they stressed that due to the potential emotional labor of such work, a willingness to assist should not be assumed by everyone. Even among those willing to educate themselves, a barrier might be finding reliable sources of information. We recommend reaching out to local and national community groups and organizations that engage trans and nonbinary people. Additional recommended educational sources include Trans Student Educational Resources (TSER; https://transstudent.org) for infographics and workshops by community members. Providers may also benefit from familiarizing themselves with, the National Center for Transgender Equality (https://transequality.org/) and engaging with the many resources made available by national LGBTQ+ healthcare providers and leaders (see for example the Fenway Health Institute at https://fenwayhealth.org). Moreover, most professional licensing and accrediting bodies require health and human service workers to engage in ongoing continuing education. In recognizing the health disparities faced by trans and nonbinary people, such organizations should consider continued efforts to develop and make available provider-focused trainings on affirming care.

**Figure 2:**
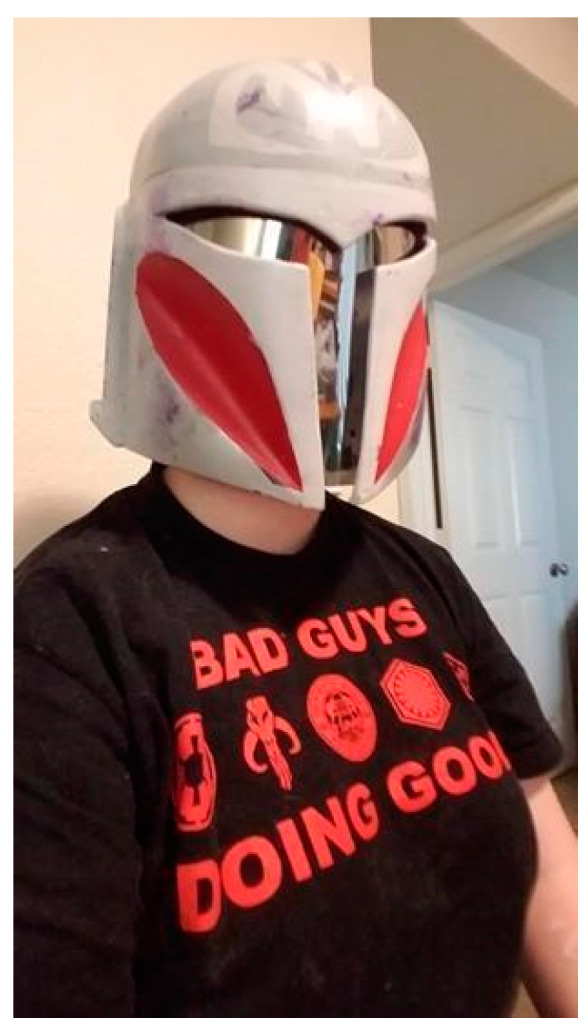
“This is my face” (Cory).

**Listen to Us, Trust Us, and Commit to Doing Better.** PRs desired people in their lives who were committed to change and engaging in their lives in affirming ways. “I feel like people don’t realize just showing up is a huge deal for most… I feel like I can just relax, and that’s such a big deal” (Kai). Relationships—both personal and professional—were reported to be pivotal in the lives of the PRs. Many factors contribute to whether these relationships hinder or help their wellbeing. PRs invited anyone who wanted to align themselves with and support nonbinary individuals in their fight for justice, equity, and liberation. PRs identified specific actions that they felt were supportive of their gender affirmation. Articulating the range of desired supports, Noel was succinct and to the point in what they hoped providers would do: “advocating, educating others, and most importantly, taking the time to listen to us. Truly listen to us”. Frequently, PRs reported that they faced people’s disbelief or suspicion regarding their nonbinary gender. Often, such suspicion resulted in people wanting (and in some cases demanding) PRs to prove their gender, regardless of their gender expression, in order to access gender-affirming hormones (e.g., facial hair) and surgery (e.g., flat chest from top surgery). In sharing Figure 2, Cory explained the pain caused when others demand personal details about someone’s gender. They explained, “Sometimes you have to accept people at face value rather than being rude and asking all sorts of reaching, invasive questions—like respect the mask rather than try and rip it off if somebody doesn’t want to share”.

Those who interact with nonbinary people were thought to play an essential role in improving the environment for nonbinary individuals across multiple settings (e.g., employment, education, medicine, mental health). Central to key individuals improving various environments for nonbinary people was the concept of trust-building. Building trust within relationships with nonbinary people was described as starting with simply believing people when they share their gender.

#### 3.5.1. Recommendations for Increasing Access to Gender-Affirming Care

**Reduce barriers.** PRs identified numerous ways in which they navigated barriers as they attempted to obtain gender-affirming medical care (inclusive of hormones and gender-affirming surgeries). Significant barriers to accessing healthcare included gatekeeping, limited health insurance coverage, and financial feasibility.

Prevalent forms of gatekeeping included a lack of knowledgeable providers and systems that explicitly excluded nonbinary individuals. As previously discussed, providers who lack knowledge about nonbinary people were reported to endorse binary gender paradigms and create exclusionary environments or outright hostile to nonbinary people. Moreover, binary paradigms have been codified with medical frameworks and diagnostic criteria related to affirming care. As Jynx described, “I can’t be out, the medical system doesn’t have a way to be nonbinary in the medical system right now”. From a lack of diverse gender options on forms to binary requirements for services, PRs reported experiencing limited opportunities for access to gender-affirming care. Paying particular attention to the pathologizing nature of gender-related diagnoses in the diagnostic and statical manual (DSM) and the binaristic criteria such diagnoses are built upon, PRs felt particularly strongly about the need to remove insurance requirements for a gender dysphoria diagnosis to access affirming healthcare. Even among PRs who experience gender dysphoria, they agreed:

*I am one of the trans people with dysphoria, but I also don’t believe you have to have dysphoria to be trans and I will fight anybody who says otherwise…I don’t think we should be forcing…diagnosis…we shouldn’t be forcing mental health problems on people because they happen to have their own sort of gender dysphoria or like gender journey.* (Jynx)

Even when access to transition-related care was available, PRs experienced “costs and other barriers to affirming processes such as name changes and surgeries” (Gabi). Considering the documented risk to mental health, providers advocating for policy change could reduce barriers to trans-affirming care and significantly improve the wellbeing of nonbinary individuals.

**Figure 3:**
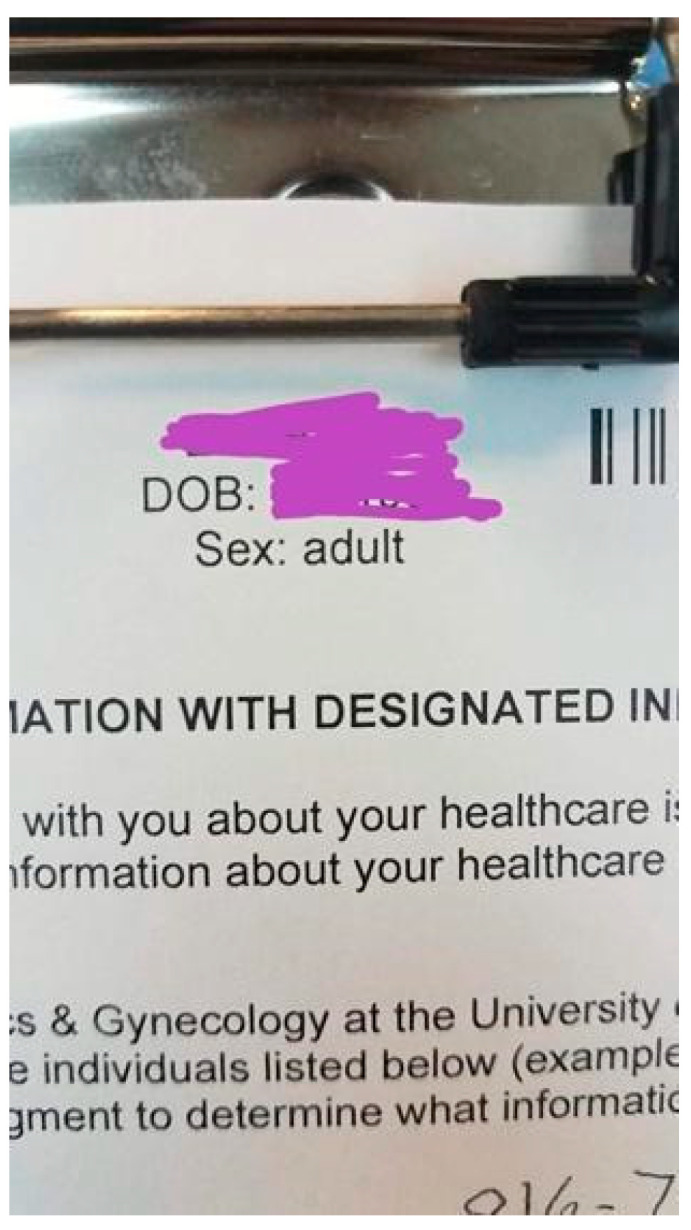
“Healthcare Euphoria” (Cory).

**Create Affirming Environments.** PRs reported being more likely to share their gender identity and expression when in an environment that did not impose assumptions on them. Creating affirming spaces in professional settings was recommended through documentation, visual cues, and facilities. At a minimum, PRs wanted to see room for chosen names and pronouns on in-take forms and other paperwork. For example, “when doctor’s offices have first-time visitors’ charts, instead of the usual ‘M’ or ‘F’ for gender, have more expansive gender options, maybe even fill-ins” (Skyler). In the absence of gender on forms, a provider attempted to be affirming as seen in Figure 3, which Cory titled Healthcare Euphoria because they appreciate the effort and felt seen. This example highlights that simple changes can have a significant impact on the lives of nonbinary individuals, or in this case the difference between dysphoria or euphoria.

**Figure 4:**
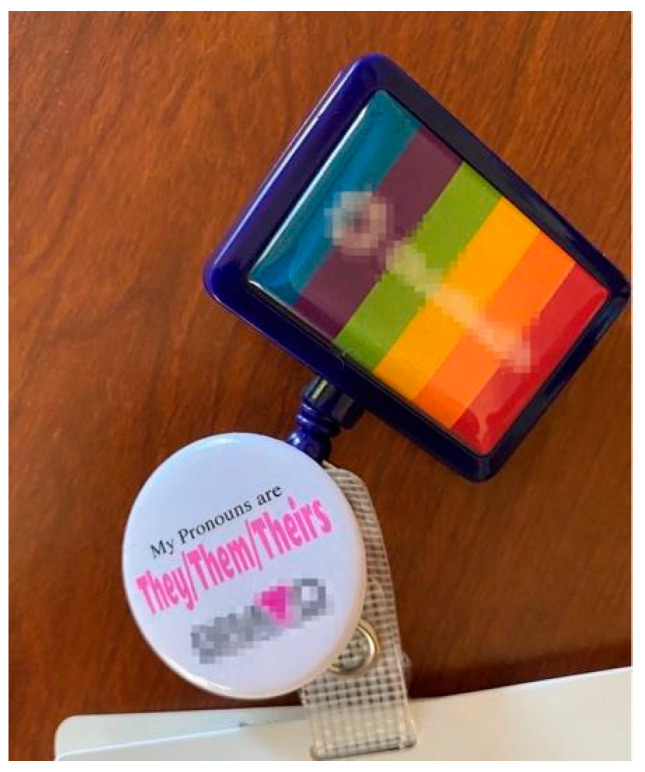
“Hi my name is [E.] and my pronouns are they/them” (E.).

In addition to inclusive documentation, other visual cues mentioned were rainbow flags, literature that included trans and nonbinary people, pronouns on providers’ name badges, and greeting patients with non-gendered language (e.g., “hello friend” or “good morning” without “sir” or “ma’am”). A resounding recommendation has been to ask for and correctly use patients’ names and pronouns. Emphasizing this point, Boots stated, “we need str8 [and cisgender] ppl to put their pronouns on their business cards and email signatures to help us normalize personal identity relevance”. Pronoun usage can be normalized throughout correspondences, websites, name badges, and sharing/asking during introductions (see Figure 4).

**Figure 5:**
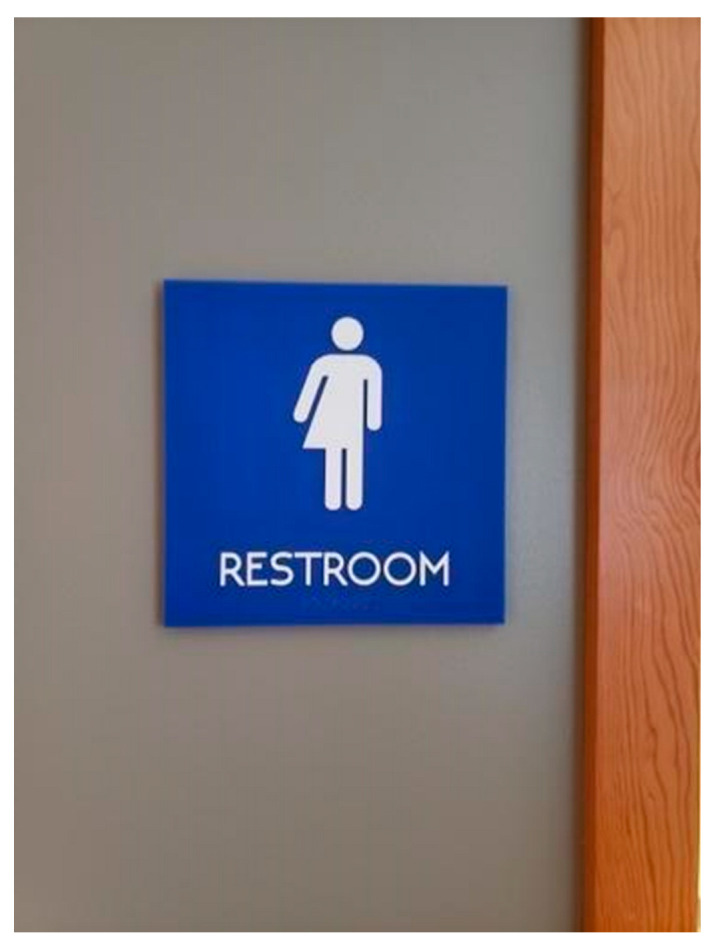
“We Don’t Have a ‘Men’s’ Bathroom” (Rowan).

A significant environmental factor that was consistent across public spaces was all-gender or single-stall bathrooms (see Figure 5). Nat shared that they faced a policy at work regarding older buildings that disallowed disability-accessible and all-gender bathrooms in buildings constructed after a specific date, which meant a long commute to the closest single-stall bathroom in another building. Alternatively, converting facilities to all-gender bathrooms was repeatedly brought up by PRs as a way of alleviating anxiety and stress. 

**Improve Research and Provider Education on Transitional Care.** PRs wanted to know more about their options for medical transition care and wanted to have knowledgeable providers with whom to discuss these options. Specifically, PRs wanted more nonbinary transitional care options, such as microdosing Estrogen similar to the increasing practice of microdosing Testosterone, which was noted as also not regularly discussed by providers. For Kai, microdosing was the best fit for them—"because I wanted to be more androgynous, my doctor set me up with a low dose of testosterone…which has been great and honestly I’ve never been happier since I’ve been on it”. The need for access to a variety of nonbinary-inclusive resources was agreed upon, particularly for making informed decisions about their gender-related care.

**Figure 6:**
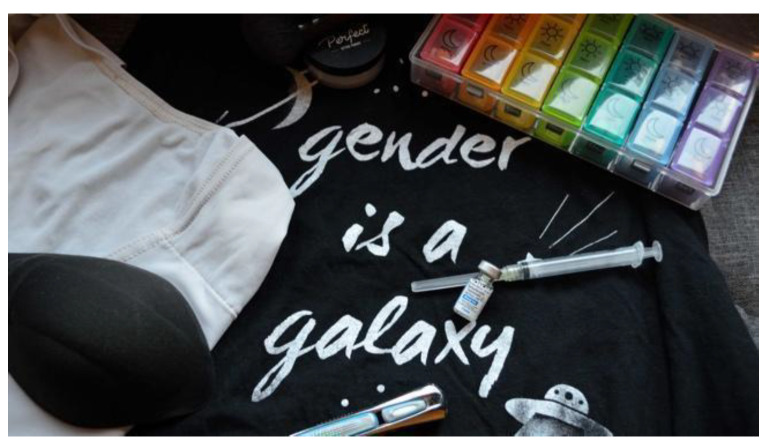
“Tools of the Trade” (Kai).

*The fact that [access to gender-affirming resources] is becoming so normalized is great and the fact that I get to have all of these tools is really incredible…[as] more people are able to get things they need to be more themselves…I can only imagine how much happier people [will be] because of that—I know I am.* (Kai) (see Figure 6)

In order to create change so that all nonbinary people can feel the fulfillment Kai described, PRs emphasized the need for resources and services to be intersectional, prioritizing gender diversity as well as racial justice, disability justice, and fat liberation, among others. Identified gaps in intersectional knowledge and affirming care were for nonbinary individuals over 50 years old, assigned male at birth, fat, and with disabilities, all of which were said to make their gender journey more challenging. Though the sample was not racially diverse, PRs were conscious of how race was a point of privilege or further marginalization. Overall, PRs wanted services that considered how these other identities intersect with gender identity and expression.

These affirming and unassuming experiences improved PRs’ access to services and the likelihood of them returning. “I can confirm firsthand from a patient side that seeing those rainbow clips [on name badges] always does make me feel safer” (PR #1). Dylan added, “visual reminders that I’m ok, that I’m safe, that who I am is celebrated also plays a role in that sense of safety”. PRs also stressed how these changes did not require much effort but made a significant difference to them. The impact of even simple acts as Cory described can send a message of affirmation. HG suggested including patients in the process of creating changes and Timothy offered a similar recommendation for the use of community advisory boards:

*I’d recommend that more LGBTQ+ and non-LGBTQ+ groups work to increase the representation of non-binary perspectives in the services and resources they provide. For instance, a mental-health-focused non-profit or resource center could actively solicit the insights of non-binary people to better provide resources to that community.*

Though other forms of education felt like emotional labor, these meaningful contributions were discussed as worth the energy because they led to direct positive impacts for themselves and their communities.

#### 3.5.2. Recommendations for Advocating for Environmental and Policy Changes

**Normalize Gender Expansiveness.** PRs recommend normalizing gender expansiveness through increasing visibility and awareness through positive, accurate, and nuanced representation of gender diversity and nonbinary people. PRs discussed the dramatically positive difference for youth growing up with nonbinary representation to help them understand their gender earlier compared to PRs as older adults coming out later in life due to the lack of information. Timothy was already seeing change—“a lot of them [students ~18–22] come in and have a really firm understanding of their identity and it’s like whoo! This is what having representation does”. These improvements in representation were said to give PRs hope for future generations.

**Advocate for Policy and Social Change.** All PRs agreed on the need for organizational policies and legislation to include protections for gender identity and expression that was inclusive of nonbinary persons. PRs called for advocacy to “ensur[e] that organizations have specific policies that work to affirm and protect nonbinary people within the organization” (Noel). A lack of protection due to non-discrimination policies that did not explicitly protect gender identity and expression was a recognized need. Dylan shared, “In Ohio, I can still be denied housing and public accommodations (thankfully not in my city/county, but still) so I’m all about nondiscrimination ordinances”. The impact of policies on their daily lives was emphasized, including employment, housing, sports, and healthcare. 

Regarding healthcare, PRs called for advocacy for structural changes to provider education. Noel shared that they wanted:

*[I]nclusive policy that creates wider access to affirming healthcare. Medicare for all is a great step, but we also need to go further and create policies that help healthcare to be more affirming and understanding of the unique issues that we face.*

Building upon Noel’s identified need for understanding of unique experiences of nonbinary individuals, Skyler wanted “doctors that don’t live by ‘male’ and ‘female’ ideals but have a broader knowledge of nonbinary genders”. A lack of such education has led PRs, such as Nat, to experience “misinformed non-trans health professionals (that try to comment on my trans healthcare)”. Nat’s quote sheds light on the importance of trans-informed professionals being needed across all healthcare, not just in trans-specific healthcare.

For some PRs, policies were thought to be a path to social changes. Just as affirming policies could increase accessibility to resources and social acceptance, un-affirming and anti-trans policies and politics were felt to delegitimize their gender and entitlement to fundamental human rights, which was described as contributing to a toxic cultural climate. Skyler noted, “Peace doesn’t come easily, especially when not only average strangers love to argue with you to senators making policies that bar you from basic activities”. Harmful public discourse had a deteriorating effect on their wellbeing. However, even when PRs felt challenges within society, policies that were inclusive and affirming helped them to feel safer and more supported in their environments. HG summarized the call for advocacy as “from the community outward to society”.

## 4. Discussion

This study built upon the existing call for trans-inclusive healthcare with nonbinary-specific care [73,74,75,76], providing community member experiences accessing healthcare and recommendations for improving gender-affirming care specific to those with expansive gender identities and expression. The PRs’ recommendations support the literature calling for client-centered gender-affirming care that promotes the comprehensive health and wellbeing of patients [1,28,35,38,73,74], while providing strategies for achieving these goals. Medical professionals have been explicitly identified as needing training about affirming nonbinary and gender nonconforming patients [28,74,77,78,79], and for that training to be intersectional in recognizing the complexity and interrelatedness of the various components of clients’ identities [73]. Not only is there a need for gender-affirming training to exist, but physicians also need to be interested in pursuing such training [80]. 

In the current healthcare system, many nonbinary people face discrimination and microaggressions that contribute to the avoidance of care [73], and that has an adverse impact on their health and wellbeing [3]. However, potential improvement in patient-provider relationships can lead to a more empowering connection through gender-affirmation care [38]. Until gender-affirming care is widespread, the need for and benefits of interdisciplinary gender health clinics continue to be crucial to positive health outcomes for trans and nonbinary people [28].

In addition to the PRs’ recommendations, the authors recommend that all providers adopt a trauma-informed approach to working with transgender and nonbinary individuals. Marginalization related to gender nonconformity has been shown to contribute to greater mental health distress and post-traumatic stress disorder (PTSD) than conforming peers [81,82] Previous traumas, PTSD, desensitization, and disassociation were mentioned by many of the PRs, with at least one PR subjected to the harmful practice of conversion “*therapy*”. Histories of trauma indicate a need for mental health and medical care equipped to address the needs of these populations. Patient-provider relationships could be improved by understanding that trauma might be a part of nonbinary patients’ experiences and how that influences interactions within healthcare interactions and settings [74,83].

The WPATH SOC 7 stated that providers should “Be prepared to support and advocate for patients within their families and communities (schools, workplaces, and other settings)” ([35] p. 167). According to Perrin et al. [84] a lack of protective policies can contribute to internalized stigma (e.g., inferiority) and increase discrimination, while inclusive protective policies are likely to promote equity and invoke community belonging. While healthcare policies are written on the binary, nonbinary individuals will continue to experience barriers to gender-affirming medical interventions. The need for advocacy is apparent.

### Study Limitations

Extraneous variables were key limitations of the study. Data were collected during the summer of 2020, which posed unanticipated and unprecedented confounding factors to the study—namely, a pandemic and a social movement that incited social unrest (For clarification, the culmination of social unrest after an increasingly widespread documentation of Black people being murdered by police, was an anticipated response, just not an anticipated factor for this study). The sample may have been biased towards self-selecting individuals with greater community connections, individual wellbeing, and representation to affirm they qualified for the study. While this study actively sought a diverse sample, several factors likely limited recruitment of the intended sample, namely a white researcher whose race may not have instilled trust among racial minorities. Related, the co-occurring social unrest around the ongoing murder of Black people may have understandably been a priority over the study and thus reduced the available time and emotional capacity of interested participants. The lack of recruiting a racially diverse sampling was likely influenced by the COVID-19 pandemic, during which LGBTQ people of color were twice as likely as their white counterparts to test positive and were furloughed and laid off at a disproportionately higher rate causing additional financial stressors during the crisis [85]. The limited diversity within the sample leaves gaps in understanding wellbeing among nonbinary individuals with other intersectional identities and poses an opportunity for needed future research with targeted populations.

## 5. Conclusions

Nonbinary individuals face barriers to healthcare due to transphobia and binary systems that adversely impact their mental and physical health. The literature supports the need for improved healthcare and the repercussions of the current interpersonal and systemic transphobia and binarism that pose barriers to standard and gender-related care. This study identified ways to bolster wellbeing among nonbinary individuals through increased access to gender-affirming healthcare. The findings shared within this article focus on reflections from nonbinary people regarding their experiences accessing healthcare and offer recommendations for improving access to such services. Key elements posited as means to improve access to and quality of care include personal accountability for interpersonal care, organizational and systemic changes to increase access to gender-affirming care, and professional advocacy for environmental and policy changes. Collectively, the results evidence a need for a shift in healthcare from personal to systems to become more cognizant of and responsive to the needs of nonbinary patients. These recommendations were created with earnestness and hope by participant researchers that providers will take action with this information to make this world more inclusive and affirming for nonbinary individuals. 

## Figures and Tables

**Table 1:** Participant Researcher Demographics (*N* = 17).

	*M*	*sd*
Age (range: 19–50)	30.00	7.40
	*f*	%
Race		
Multiracial ^1^	1	5.9
White	16	94.1
Ethnicity		
Eskinazi	1	5.9
German Irish	1	5.9
Native American	1	5.9
Non-Hispanic	3	17.6
Non-Latinx	1	5.9
White	5	29.4
“White-washed”	1	5.9
Chose not to respond	4	23.5
Current Gender (multiple)		
Agender	2	11.8
Genderfluid	2	11.8
Gender-neutral	1	5.9
Genderqueer	5	29.4
Nonbinary	12	70.6
Transgender	1	5.9
Transmasculine	2	11.8
Pronouns ^2^ (multiple)		
He/him/his	3	17.6
She/her/hers	2	11.8
They/them/their	17	100.0
Assigned sex at birth ^3^		
Assigned female at birth	10	58.8
Assigned male at birth	1	5.9
Intersex	1	5.9
Chose not to respond	5	29.4
Identify as disabled/someone with a disability ^4^	9	52.9
Identify as neurodiverse/someone with neurodiversity ^5^	7	41.2

Note: Demographic categories are listed alphabetically to avoid biased ordering. PRs were given the option to answer with more than one label, reported by frequency for current gender and pronouns. ^1^ Multiracial—Puerto Rican and Indigenous. ^2^ Pronouns—Notably, none of the PRs in the final sample used neo pronouns; however, two PRs who withdrew before starting used the neo pronouns xie/xex/xyr and zee/zed/zeta. ^3^ ASAB—Assigned sex at birth. As one PR noted, “leave off—that is the first thing people immediately jump to”. Of the eight PRs who withdrew before or at the start of the study, at least three were AMAB. ^4^ Disability—(yes reported). As disability can mean many things, descriptions included chronic pain, Scoliosis, AGDS, visual disability, and tinnitus. One PR reported a medical condition but did not consider it disabling even though it might be considered a disability. ^5^ Neurodiversity—(yes reported). Again, neurodiversity is an umbrella category, therefore, we have included their descriptions of anxiety, depression, ASP, ADHD, BPD, “not neurotypical”, “among others”. One PR reported they do not apply the label to themselves but have anxiety disorder and PTSD.

## Data Availability

The data presented in this study are available on request from the corresponding author. The data are not publicly available due to the privacy of participants, who belong to a marginalized population.
